# Dr. Thomas B. Cosford

**Published:** 1904-04

**Authors:** 


Dr. Thomas B. Cosford, of Lockport, died February 26, 1904, at Redlands, Cal., aged 58 years. He was for many years a prominent physician of Lockport. Failing health led to a visit to California last winter, but it did not stay his disease.

